# First Evidence of Fowl Adenovirus Induced Inclusion Body Hepatitis in Chicken in Bangladesh

**DOI:** 10.1155/2023/7253433

**Published:** 2023-01-03

**Authors:** Mohammad Nazrul Islam, Md. Mostafizer Rahman, Md. Khalesur Rahman, Jahangir Alam

**Affiliations:** ^1^Department of Microbiology, Faculty of Veterinary and Animal Science, Hajee Mohammad Danesh Science and Technology University, Basherhat, Dinajpur, Bangladesh; ^2^Animal Biotechnology Division, National Institute of Biotechnology, Ganakbari, Ashulia, Savar, Dhaka, Bangladesh

## Abstract

**Background:**

The livestock sector contributes 1.90% to the GDP in Bangladesh during 2021–22. Poultry is one of the important subsectors struggling with diseases. Fowl adenoviruses (FAdVs) cause numerous diseases resulting in economic losses to the poultry industry worldwide. Several FAdV serotypes cause inclusion body hepatitis in chicken. Although FAdV infection was suspected, there was no confirmatory report from Bangladesh. The study was conducted to investigate the FAdV infection and antibodies in chicken.

**Methods:**

A total of 50 samples, each composed of liver and spleen, were collected from different chickens of Gazipur, Dinajpur, and Panchagarh district. Each location belongs to A, B, and C poultry zones of Bangladesh, respectively. Viruses were detected by real-time PCR and conventional PCR. Blood samples (*n* = 303) were collected at the beginning and after the recovery from infection and tested by indirect ELISA. Sequencing of PCR products was done for serotyping and phylogenetic analysis.

**Results:**

Clinical signs were observed including anorexia, drowsiness, ruffled feathers, reduced body weight, lack of uniformity, and high mortality (15–25%). Enlarged friable liver with yellow to tan color mottled with the focal soft area, fluid in pericardial sac, swollen and hemorrhagic kidneys, enlarged congested spleen and pancreas, etc. were found on postmortem examination. FAdVs were detected in 90% of the flocks except commercial layer flock from Dinajpur. Three serotypes, namely, 8b (70%), 11 (10%), and 5 (10%) were detected. Anti-FAdV antibody was detected in 80% flocks at the beginning of infection and in 90% of the flocks after recovery from infection. The antibody titer increases significantly (*p* < 0.05) after recovery from infection. Phylogenetic analysis revealed that the Bangladeshi FAdVs have close identity with viruses from Asia, Europe, and South and North America.

**Conclusions:**

These findings suggested that several introductions of FAdVs were taken place in Bangladesh. To combat the disease, vaccination along with maintenance of biosecurity is essential.

## 1. Introduction

Adenoviruses (AdVs) are nonenveloped, double-stranded DNA viruses belonging to the family Adenoviridae consisting of five genera, namely, Mastadenoviruses, Aviadenoviruses, Atadenoviruses, Siadenoviruses, and Ichtadenoviruses [1]. Fowl adenoviruses (FAdVs), with a genome size of 43–45 kb [2] are composed of three groups (I–III) [3] and five different species, namely, FAdV-A to FAdV-E based on restriction fragment length polymorphism (RFLP) [4]. Furthermore, they are classified into twelve serotypes such as FAdV-1 to FAdV-8a and FAdV-8b to FAdV-11 based on cross-neutralization tests [5, 6]. FAdV-A and FAdV-B contain serotypes 1 and 5, respectively. Serotypes 4 and 10 are included in FAdV-C. Serotypes 2, 3, 9, and 11 belong to FAdV-D, whereas serotypes 6, 7, 8a, and 8b are included in FAdV-E [7].

FAdVs cause a variety of diseases in chicken, and inclusion body hepatitis (IBH), hydropericardium hepatitis syndrome (HHS), and gizzard erosion and ulceration are most important of them. FAdV-1 of species A and FAdV-4 of species C are the causative agents of HHS and have been isolated from most cases of gizzard erosion and ulceration [8, 9]. IBH is an acute disease caused by FAdV-D serotype 2, 3, 9, and 11 and FAdV-E serotype 6, 7, 8a, and 8b [10, 11]. The virus is transmitted vertically as well as horizontally [12], distributed throughout the world [13], and causes infection mainly in broiler chickens of 3–7 weeks of age, resulting sudden increase of mortality which occasionally might be as high as 30% [14–16]. IBH outbreaks have been confirmed in different countries and serotypes 2, 4, 8a, 8b, and 11 have been reported as the most frequent causal agents [17–19]. The pathogenicity of recently isolated strains, mainly of FAdV-8b and 11 have been investigated with inconsistent results. Some studies had shown less to severe clinical signs and mortalities when infecting chickens of different ages, like from 1 day to 3 weeks of age, with FAdV serotypes 8b and 11 [20, 21].

Phylogenetic analyses of partial *hexon* gene sequences are an adequate and quick method for differentiation and genotyping of FAdVs [22]. Polymerase chain reaction (PCR) with primer sequences based on the *hexon* gene or the 52 K gene is useful for virus identification [23–26]. Besides, PCR together with DNA sequencing and/or restriction enzyme analysis has been used for FAdVs typing [27]. Real-time PCR and subsequent high-resolution melting (HRM) curve analysis of three regions of the *hexon* gene were also reported and assessed for their potential in differentiating 12 FAdVs reference serotypes [28]. Also, a SYBR green-based real-time polymerase chain reaction is reported for detection and quantification of all FAdVs [26].

Bangladesh is largely an agricultural country and livestock is an integral part of the complex farming system. Livestock is not only a source of meat protein but also a major source of farm power services as well as employment. The livestock subsector provides full time employment for 20% of the total population and part-time employment for another 50% [29]. Poultry is a rapidly growing sector, and its expansion is being driven by the rising incomes and shift in industry structure. In Bangladesh, there are roughly 30.41 million chickens, and poultry farms are expanding at a pace of 15% annually. Poultry meat accounts for a sizable 37% of Bangladesh's total meat production [30]. At constant prices, the contribution of livestock sector to the GDP in the financial year 2021–22 was 1.90% and the contribution of livestock to the overall agricultural sector was 16.52 percent [31]. The greatest impact of poultry in sustainable development goal designed to help the poor is enhancement of livestock-production systems [32]. However, the poultry farmers still facing several challenges including disease outbreak causing huge loss in poultry production due to high morbidity and mortality [33]. Outbreak of several diseases was like Newcastle disease (ND), infectious bursal disease (IBD), infectious bronchitis (IB), egg drop syndrome (EDS), and fowl cholera (FC), etc. occurred every year, resulting enormous economic losses [34].

Based on the results of an agar gel diffusion test FAdV infection of broiler parents was reported from Bangladesh in 2002 [35]. However, no one reported the prevalence of this disease based on molecular analysis. Farmers and veterinary professionals in Bangladesh have noticed clinical signs including unexpected mortality, slower growth, and greater feed conversion ratio over the past few years, and they assume the IBH virus may be the cause [33]. But none of the suspected cases were confirmed by laboratory tests including molecular technique [33]. The disease is thought to have invaded Bangladesh and is currently spreading throughout the poultry industry, especially among broiler chickens. Investigating the disease-causing agent and developing preventative measures are thus necessary. In this study, we have evaluated and reported the FAdVs in suspected chickens from three geographically distinct regions in Bangladesh using clinical, virological, and serological methods. This is the first report to date that shown virological including sequencing and phylogenetic analysis as well as serological evidences of FAdV infection in chicken in Bangladesh.

## 2. Materials and Methods

### 2.1. Study Design and Ethical Approval

This cross-sectional study was conducted during November 2020-August 2022. For this study tissue samples were collected from dead birds. However, blood samples were collected from live birds. Before collecting blood, owners' verbal consents were obtained. The Hajee Mohammad Danesh Science and Technology University's Institute of Research and Training granted ethical approval for this study under the number HSTU/IRT/2930(1).

### 2.2. Study Area

**Figure 1:**
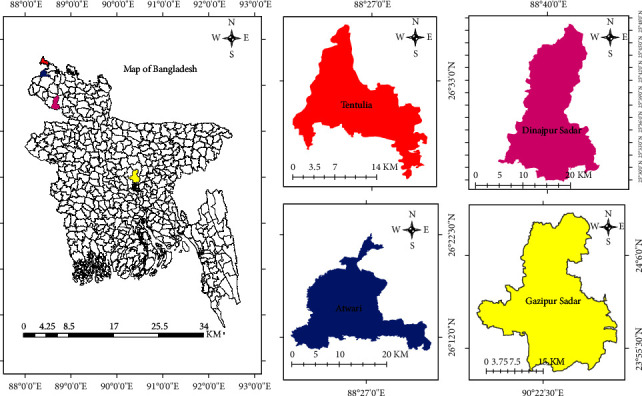
Location of the sampling sub-districts (Upazila) of Bangladesh marked by different colors. Map is created in ArcGIS 10.8.2. ArcGIS Enterprise, ESRI, Redlands, California, USA.

In Bangladesh, chickens are available over the entire nation. Huque and Khan [36] riven the country into three zones: A (105–1212 chickens per square kilometer), B (344–1108 chickens per square kilometer), and C (252–868 chickens per square kilometer) based on the density of chickens. This study includes Dinajpur Sadar upazila of Dinajpur district from zone A, Tetulia and Atwary Upazilas of Panchagarh district from zone B and Gazipur Sadar Upazila of Gazipur district from zone C (Figure 1).

### 2.3. Collection of Samples

A total of ten flocks of five different farms located in geographically distant three locations viz. Gazipur Sadar Upazila, Dinajpur Sadar Upazila, Tetulia, and Atwar Upazilas of Panchagarh district, were included in this study. The flocks include one commercial layer flock (5000 birds) from Dinajpur district, four broiler breeder flocks (10,000 birds/flock) from Panchagarh district and three broiler flocks (2000 birds/flock) and two broiler breeder flocks (10,000 birds/flock) from Gazipur district (Table 1 and Figure 1). From each flock five samples were collected. Each sample composed of liver (∼2 gm) and whole spleen of a suspected dead chicken. So, from ten flocks a total 50 samples were obtained for virological investigation. Besides, 303 blood samples, 156 samples at the beginning of suspected infection and 147 samples after recovery from suspected infection were collected from these ten flocks to check the anti-FAdV antibody titer (Table 2).

### 2.4. Nucleic Acid (DNA) Extraction

Tissue samples, consisting of liver and spleen, were homogenized in phosphate-buffered saline (pH 7.0–7.4) with 1 : 10 w/v ratio. The homogenates were kept at −80°C for 2–3 hours and thawed at room temperature for ∼30 min. Freezing and thawing cycle was repeated for three times and finally centrifuged at 8000 × *g* for 3 min. Supernatant was collected and used for DNA extraction. PureLink genomic DNA Mini Kit (Invitrogen, USA) was used to extract DNA as per manufacturer's instruction. The purity of the extracted DNA was checked by NanoDrop™ 1000 Spectrophotometer (Thermo Fisher Scientific, USA) and preserved at −20°C until further analysis.

### 2.5. Detection of FAdVs Molecular Test

Samples were principally tested by realtime PCR (Rt-PCR). However, some samples were also tested by conventional PCR. Rt-PCR was also done for serotyping of FAdV serotype-4 and 8b. For Rt-PCR, Taqman chemistry based FAdV Pockit kit (GeneReach Biotechnology Corp, Taiwan) was used for detection of FAdV-A to FAdV-E and FAdV-4 and FAdV-8b serotype specific kits were used for serotyping. Rt-PCR was carried out on a 7500 FAST Real-Time PCR System (Applied Biosystems) with ABI 7500 software (Version 2.3). The reaction was set in 25 *μ*L scale and consisted of 2 *μ*L template DNA and 23 *μ*L of premix reagents. The thermal profile consisted of the 40 cycles of 3 min at 95°C, 15 sec at 93°C and 1 min at 60°C.

In case of conventional PCR, fragment of loop 1 (L1) region of the *hexon* gene of FADV was amplified by using the primers- F: 5′-ATGGGAGCSACCTAYTTCGACAT-3′ and R: 5′-AAATTGTCCCKRAANCCGATGTA-3′ described earlier [37]. The PCR reaction mixture was in volume of 25 *μ*L containing 12.5 *μ*L of master mix (Thermo Scientific, Waltham, MA, USA), 2 *μ*L (10 *μ*M) of each forward and reverse primer, 6.5 *μ*L of nuclease free water and 2 *μ*L of template DNA. Negative control, all reagents except test sample, was run along with test sample and total volume was adjusted with nuclease free water. Amplification was carried out in a 2720 thermal cycler (Applied Biosystems, UK). The thermal profile was 10 min at 95°C for initial denaturation followed by 40 cycles of denaturation at 95°C for 15 sec, annealing at 56°C for 30 sec, and extension at 72°C for 45 sec, and a final extension at 72°C for 5 min. Amplicons were analysed in ultrapure 1.5% agarose gel (Invitrogen), containing SYBR Safe DNA gel nucleic acid stain (Thermo Fisher Scientific) using 1X TBE electrophoresis running buffer.

### 2.6. Sequencing and Phylogenetic Analysis

Sequencing was done with Applied Biosystems 3130 Genetic Analyzer using 20 *μ*L reaction volume containing approximately 10 ng purified PCR product as template, 5X ready reaction premix 4.0 *μ*L, Big Dye terminator buffer 2.0 *μ*L, primer 0.32 *μ*L and ultrapure water to make 20 *μ*L. The primers used for amplification was also used for sequencing. The sequence was read from both directions. The electropherogram analysis and multiple sequence alignment (MSA) were done using MEGA-11 software [38]. The evolutionary history was inferred using the Neighbor-Joining method and Tamura–Nei model [39]. Initial tree for the heuristic search were obtained automatically by applying Neighbor-Join and BioNJ algorithms to a matrix of pairwise distances estimated using the Tamura–Nei model, and then selecting the topology with superior log likelihood value. Evolutionary analyses were conducted in MEGA11 [38].

### 2.7. GenBank Accession Number

Nucleotide sequences generated in this study were submitted to GenBank and obtained accession number (Table 1).

### 2.8. Detection of Anti-FAdV Antibody by Indirect ELISA

Serum samples were investigated for the detection of antibody to FAdV by indirect ELISA using a commercial FAdV Group-1 antibody test kit (Biochek, Reeuwijk, Holland) following the manufacturer's instructions. Initially, 5 *µ*L serum was mixed with 245 *µ*L of sample diluent which gives serum a dilution of 1 : 50. Then, 50 *µ*L diluted serum was added in to the antigen coated palate having 50 *µ*L sample diluent (1 : 100) except two negative and two positive control wells. About 100 *µ*L/well positive and negative control was added. The plate was covered with lid and incubated at room temperature (22–27°c) for 30 minutes. Then, the mixture of each well of the plate was aspirated and washed four times with wash buffer (350 *µ*L per well). The plate was the inverted and tap firmly on absorbent paper to remove residual wash buffer. Then, 100 *µ*L of conjugate was added and incubated at room temperature (22–27°c) for 30 minutes after covering with lid. Up on incubation, the contents of wells were aspirated and washed four times with wash buffer (350 *µ*L per well). Residual buffer was removed as mentioned earlier. Then, 100 *µ*L of substrate was added and incubated at room temperature (22–27°c) for 15 minutes after covering with lid followed by addition of 100 *µ*L of stop solution into each wells. The absorbance value was taken at 405 nm. The sample/positive (*S*/*P*) ratio was employed using following formula to proceed for interpretation of results.

(1) $$\begin{array}{c} \frac{S}{P}=\frac{Mean\;of\;each\;test\;sample-Mean\;of\;negative\;control}{Mean\;of\;positive\;control-Mean\;of\;negative\;control}\text{.} \end{array}$$

Moreover, the following equation was used to relate the *S*/*P* of a sample at 1 : 100 dilutions to an end point titer.

(2) $$\begin{array}{c} \mathrm{Log}10\;Titer=1.1*\mathrm{Log}10\left(\frac{S}{P}\right)+3.361\text{.} \end{array}$$

Samples with an *S*/*P* ratio of 0.5 or greater were considered as positive for anti-FAdV antibody. In other words, samples having titer 1071 or greater are considered as positive.

### 2.9. Statistical Analysis of Data

The Statistical Package for Social Science (SPSS) version 20 was used to analyze the collected data. The descriptive results were presented as mean and standard error. Unpaired *T* test was used for two variables (BE: Beginning of Exposure and AE: After Exposure). The differences were considered statistically significant at *p* values < 0.01 or < 0.05.

## 3. Results

### 3.1. Clinical Findings

**Figure 2:**
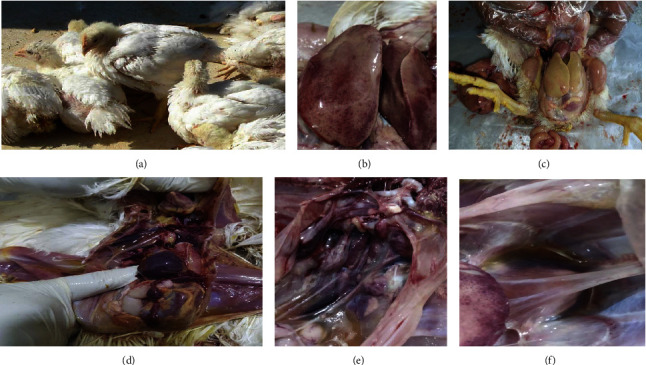
Clinical signs and postmortem lesions of chicken infected with fowl adenoviruses. (a) Depression, ruffled feather & reluctant to move of 1.3 wks broiler breeder, (b) hemorrhagic, pale and enlarged liver of 7 days old broiler, (c) tan colored liver of 7 days old broiler, (d) swollen and congested spleen of 15 days old broiler breeder, (e) swollen and hemorrhagic kidney of 10 days old broiler breeder, and (f) hydropericardium of 12 days old broiler.

Ten flocks of broilers (*n* = 3), broiler breeders (*n* = 6), and commercial layer (*n* = 1) from five farms of three geographically distant locations, Gazipur, Dinajpur, and Panchagarh districts, of Bangladesh were investigated. Veterinary practitioners and farmers reported that they observed drowsiness, ruffled feathers, high mortality, etc. in their farms and taken veterinary care with history of no response to treatment even with different antibiotics. Broiler flocks B1, B2, and B3 were derived from one farm and broiler breeder flocks BB1 and BB2 were derived from another farm in the Gazipur district. On the other hand broiler breeder flocks BB3, BB4 and BB5, BB6 were originated from the Atwary and the Tetulia Upazila, respectively, in the Panchagarh district. Distances among the multiple flocks of each of the farms were within 50–80 feet. Only single commercial layer flock (CL01) derived from Dinajpur Sadar Upazila. Clinical signs were like anorexia, depression, reluctant to move, drowsiness, reduced body weight gain, lack of uniformity, respiratory distress, etc. in chicks of up to five weeks of age were also noticed during farm visit (Figure 2). Among the studied flocks (*n* = 10) morbidity was found 10%–65% whereas mortality varies from 5% to 30%. However, morbidity and mortality in FAdV positive flocks were within 23%–65% and 12%–30%, respectively. Higher morbidity (65%) and mortality (30%) was found in a broiler breeder flock of Tetulia Upazila, Panchagarh, and later serotype 11 was detected from that flock. Flocks affected with serotype 8b had 23%–45% morbidity and 15%–25% mortality among the flocks. Lowest morbidity (10%) and mortality (5%) was found in the commercial layer chicken flock of Dinajpur Sadar. However, FAdV was not detected from this flock. Comparatively, higher morbidity (35%–65%) and mortality (12%–30%) was found in broiler breeder flocks than broiler flocks where morbidity 25%–30% and mortality was 15%–20%, respectively. The mortality reduced gradually 2–3 weeks later.

### 3.2. Postmortem Lesions

Hemorrhages at skeletal muscle, an enlarged, friable liver with a yellow to tan color that was mottled with a localized soft area, and even petechial and ecchymotic hemorrhages beneath the capsule and parenchyma were discovered during the postmortem examination. Accumulations of fluid in pericardial sac were also found. Swollen and hemorrhagic kidneys were frequent, and the enlarged congested spleen and pancreas were also noticed. Figure 2 shows the representative images of postmortem lesions of different ages of chickens died of infection of FAdVs.

### 3.3. Prevalence of FAdVs

**Figure 3:**
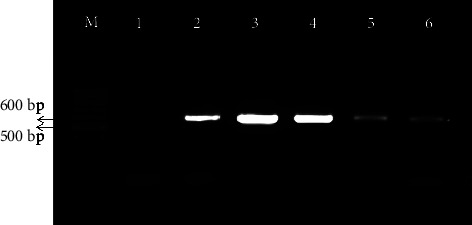
Amplification of loop 1 (L1) region of hexon gene of fowl Adenoviruses. Lane M 100 bp DNA ladder (Gene Ruler, Thermo Scientific), Lane1: negative control. Lane 2–6: test sample.

FAdVs were investigated in fifty samples originated from different broiler flocks (*n* *=* 3), broiler breeder flocks (*n* *=* 6) and commercial layer flock (*n* *=* 1) from Gazipur (*n* *=* 5), Dinajpur (*n* *=* 1), and Panchagarh (*n* *=* 4) districts of Bangladesh. At farm level the prevalence of FAdVs was 80% (4/5), whereas prevalence was 90% (9/10) at flock level. By using Rt-PCR, 37 out of 50 samples were confirmed to be positive for FAdVs, representing a 74% prevalence rate (Table 1). Some of the samples were also tested by conventional PCR. By conventional PCR, ∼600 bp DNA fragment from loop-1 region of the *hexon* gene was amplified and a representative image is shown in Figure 3. There was complete agreement between the two tests' results (data not shown). Viruses were detected in samples of all broiler and broiler breeder flocks collected from Gazipur and Panchagarh districts of Bangladesh. Ages of virus positive chickens were within 7–15 days. FAdV could not be detected in samples of commercial layer flock collected from Dinajpur district.

### 3.4. Circulating FAdV Serotypes

Rt-PCR with specificity for serotype 4 and serotype 8b was used. Besides, sequencing of loop1 region of *hexon* gene amplified by conventional PCR was also done for serotyping of FAdV. Three serotypes of FAdV viz. FAdV-5 of species B, FAdV-8b of species E and FAdV-11 of species D were detected. FAdV-8b was found in seven flocks (B1-B3, BB1-BB4) out of nine positive flocks, while FAdV-11 was found in two flocks (BB5, BB6) and FAdV-5 in one flock (BB1). FAdV-5 and FAdV-8b serotypes were detected from Gazipur district, while FAdV-8b and FAdV-11 serotypes were detected from samples of Panchagarh district. Serotype 8b was detected from samples of Atwary upazila while serotype 11 was detected from Tetulia upazila (Table 1) of Panchagarh district. Coinfection with serotype 8b and 5 was found in one broiler breeder flock (BB1) of Gazipur district.

### 3.5. Phylogenetic Analysis

**Figure 4:**
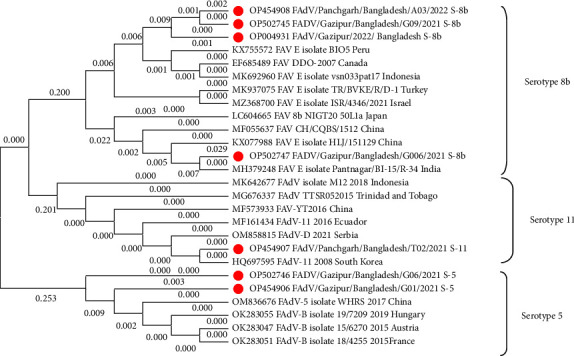
Evolutionary relationships of fowl adenoviruses. Evolutionary analyses were conducted in MEGA11. The evolutionary history was inferred using the neighbor-joining method. The evolutionary distances were computed using the maximum composite likelihood method and are in the units of the number of base substitutions per site. Viruses sequenced in this study were marked with red circle.

Evolutionary analyses of FAdVs were conducted in MEGA11 using the Neighbor-Joining method. The viruses were separated into three distinct clusters representing three different serotypes of FAdVs (Figure 4). In the tree Bangladeshi FAdV serotype 8b, serotype 11 and serotype 5 were clustered separately with other viruses. About 99.91% nucleotide identity was found among Bangladeshi FAdV serotype 8b. On the other hand, 99.99% homology was found between two FAdV-5 serotypes. FAdV serotypes 5 of this study were found to have 100% homology at amino acid level. Similarly, 100% homology was also found in case of serotypes 8b. However, the distance between the serotype 5 and 8b was found 0.42%, between the serotypes 5 and 11 was 0.39% and between the serotypes 8b and 11 was 0.29%. Phylogenetic analysis revealed that the Bangladeshi FAdVs obtained from chicken has close identity with viruses from Asian (India, China, Indonesia, Korea), European (Austria, France, Hungary, Serbia) South American (Peru, Ecuador) and North American (Canada) countries. Bangladeshi FAdV serotype 8b has close relation with virus sequences from Peru (KX755572, 2015), Canada (EF685489, 2007), India (MH379248, 2018), Indonesia (MK692960, 2017). On the other hand serotype 11 was found to have close relation with virus reported from South Korea (HQ697595) in 2008. Besides, our serotype 11 have also clustered with viruses from Serbia (OM858815, 2021), Ecuador (MF161434, 2016) and so on. Additionally, Bangladeshi serotype 5 have clustered with viruses from China (OM836676, 2021), Hungary (OK283055, 2019), Austria (OK283047, 2015) and France (OK283051, 2015) isolated since 2015.

### 3.6. Anti-FAdV Antibody Prevalence

Anti-FAdV antibody was investigated by indirect ELISA in serum samples collected at the beginning of suspected infection (*n* *=* 156) and after recover from suspected infection (*n* *=* 147) and results are presented in Table 2. Irrespective of sampling time, the anti-FAdV antibody was found in 80% (8/10) and 90% (9/10) flocks at beginning of infection and after recovery of infection, respectively. Samples originated from commercial layer flock of Dinajpur Sadar upazila was found negative for anti-FAdV antibody. At beginning of infection only 0–40% samples were found seropositive and their titer was very low (399 ± 81–885 ± 172). On the other hand after recovery of infection 53.33–100% samples were found seropositive and their titer was found very high and ranges from 1246 ± 172–9699 ± 832. The proportional seroprevalence at sample level was higher (82.31%; 131/147) in case of samples obtained after recovery from suspected infection than that of samples taken at beginning of infection (16.67%; 26/156). The anti-FAdV antibody titers differs significantly at 99% confidence level (*p* < 0.01) in one flock and at 95% confidence level (*p* < 0.05) in eight flocks at beginning of infection and after recovery from infection (Table 2).

## 4. Discussion

We have investigated three types of chicken-namely (i) Broiler, (ii) Broiler breeder and (iii) Commercial layer chicken. It is to be noted that usually broilers are reared in open-sided house with high density of birds. Generally crumble type feed is provided at starter stage and pellet at grower and finisher stage. Biosecurity measures in broiler farms are limited and farmed very close to locality, highway, other commercial poultry operations, etc. On the other hand, broiler breeders are reared in controlled sheds and crumble feed is given from starter stage to whole production period. The farm area is isolated from highway and other poultry operations. All in and all out system is usually followed with comprehensive biosecurity measures. In case of commercial layer, birds are reared in cage with crumble feed as starter and mash feed in production period. The farm area is usually isolated from highway and other poultry operations. Moderate biosecurity measures are ensured.

The present study showed that FAdV infections have affected various ages of chickens with different clinical signs. However, clinical signs gradually declined after 2–3 weeks of first appearance. The virus might be transmitted by horizontally and/or vertically. We could not determine whether the route of virus transmission was vertical or horizontal. Mortality during IBH outbreaks may peaks within 3–4 days of clinical infection and can reach 10% and occasionally be as high as 30% [11, 18, 19, 21]. Our findings of 15–25% mortality, clinical signs and postmortem lesions (Table 2) are in compliance with the findings of above studies. Moreover, IBH induced 100% mortality in commercial broiler chickens was reported from Malaysia in 2005 [40]. We have found serotype 8b of genotype E as the most prevalent FAdV in studied chicken population in Bangladesh. Circulating other two serotypes included FAdV 5 and 11. It is reported that during last ten years, the most reported IBH-induced serotypes belong to species D and E [41]. Several other studies also reported that IBH outbreaks have been confirmed in different countries and serotypes 2, 4, 8a, 8b, and 11 was the most frequent serotype as a causal agent [15–17]. Serotype 11 of species E was found to cause higher morbidity and mortality in the present study. This finding is also in line with the reports of Matos et al. [42] who reported that birds infected with FAdV serotype 8b and 11 showed more severe clinical signs and moralities than those inoculated with FAdV serotypes 2, 7, and 8a. Though clinical signs like anorexia, reluctant to move, postmortem lesions viz. enlarged liver and mortality (5%) were found in commercial layer flock, we could not detect FAdV from this group of chicken. Even they were serologically negative at beginning of clinical sign or infection and after recovery from infection. These findings suggest that other pathogen may be responsible for inducing those pathological conditions. Coinfection with FAdV serotype 8b and 5 was found in flock BB2. FAdV serotype 5 was detected from flocks BB1 and BB2 of the same farm and the distance of the sheds of these two flocks was only 50 feet. We could not detect serotype 5 from samples of other flocks. So, there is no chance of contamination with serotype 5 from other flocks. Additionally, there was little chance of laboratory contamination with serotype 8b. Because, the samples of flock BB2 were collected and tested in December 2021. There were no other samples collected and tested in the laboratory at nearby dates of December 2021. Hence, this coinfection was not due to contamination and laboratory originated. In a recent study, Liu et al. [43] showed coinfection with FAdV serotypes 4 and 8b. They reported similar clinical symptoms, mortality rates and degree of tissue lesions in coinfected birds as in single infection with serotype 4. However, they found that co-infection with FAdV-4 and FAdV-8a suppresses the replication and proliferation of FAdV-4 but enhances the replication and proliferation of FAdV-8a in the chicken liver. These means co-infection may worsen the clinical conditions of chicken.

Anti-FAdV antibody was detected in 80% of the flocks at the beginning of infection and the ages of birds were within 7–16 days (Table 2). It indicates that this antibody is not due to exposure of the sampled birds to FAdVs. This antibody might be derived from mother. Exposure of parent birds to circulating field FAdVs or use of FAdV vaccine either in combination with other vaccine or as single vaccine in parents might induced antibody in them and later passed to the chicks through egg yolk. Because Bangladesh does not formally use the FAdV vaccination. Antibody titer level was less at the beginning of infection and the titer increases significantly (*p* < 0.05) after recovery from infection (Table 2) is very usual findings. Because exposed birds might develop high level of antibody after exposure. We were unable to determine whether the antibody is against serotype 8b, 11, or 5 due to the fact that we employed an indirect ELISA kit that only detects antibodies to Group-1 FAdVs and not specific serotypes. We were unable to detect antibodies in the serum of samples taken from layer flocks in the Dinajpur district, even after they had supposedly recovered from an infection. This result agrees with the results of the virus detection. Thus, additional causes may be responsible for the clinical signs, morbidity, and mortality seen in this layer flock.

Phylogenetically, Bangladeshi FAdVs were distinctly branched in to three clusters of serotypes 8b, 5, and 11 with viruses from Asian, European, and North American and South American countries of the world (Figure 4). Hence, it is assumed that multiple introduction of FAdV in Bangladesh may be happened. High homology among the serotypes may also suggest spread of viruses within the country. One FAdV-8 serotype of ours has been linked to an Indian virus (Figure 4) and the virus was detected in the sample of broiler breeder flock BB2. Bangladesh's poultry industry was formed by the importation of chickens, eggs, feed materials, utensils, etc. from several countries where FAdVs were recorded. The introduction of FAdVs in Bangladesh may be facilitated by the import of such materials. The possibility of vertical transmission may is also existed because it is well known that FAdVs can be spread vertically through the embryonated egg and horizontally [12]. Strategies should be developed to tackle the disease as different FAdV serotypes are circulating in Bangladesh and infecting chicken. One of the control strategies may be vaccination of chicken with FAdV vaccine. In addition to vaccination, maintenance of farm biosecurity is essential to reduce the incidence of infection. We could not proceed for virus isolation, cytopathic effect (CPE) observation, histopathological studies of affected organs, etc. are the limitations of this study.

## 5. Conclusion

This study provided molecular proof that three FAdV serotypes were spreading in Bangladeshi poultry. Additionally, serological evidence was presented. Our results imply that FAdVs might be newly emerging pathogens. Preventive measures like immunization and the maintenance of strong biosecurity should therefore be taken into account in the fight against the disease.

## Figures and Tables

**Table 1:** Clinical signs, postmortem lesions and molecular detection of fowl adenoviruses in tissue samples (*n* *=* 50) obtained from ten different flocks of chicken.

Flock type	Flock size	Location	Age (days)	Sampling time	Morbidity/Mortality (%)	Clinical signs observed	Postmortem lesions	FAdVwith serotype	GenBank accession number
Broiler (B1)	2,000	Gazipur Sadar	9	June 2021	25/15	Depression, Reluctant to move	Enlarged hemorrhagic liver, swollen kidneys	5/5^*∗*^ S-8b	OP004931
Broiler (B2)	2,000	Gazipur Sadar	7	June 2021	23/20	Drowsiness, ruffled feathers	Tan colored liver, swollen spleen & kidneys	5/5 S-8b	OP502745
Broiler (B3)	2,000	Gazipur Sadar	11	June 2021	30/16	Drowsiness, less body weight gain, respiratory distress	Hemorrhagic liver, hydrocardium, swollen kidneys	5/5 S-8b	—
Broiler breeder (BB1)	10,000	Gazipur Sadar	14	December 2021	35/25	Drowsiness, lack of uniformity	Enlarged liver with hemorrhage, swollen kidneys & spleen	2/5 S-5	OP454906
Broiler breeder (BB2)	10,000	Gazipur Sadar	9	December 2021	40/20	Drowsiness, ruffled feathers reluctant to move	Hemorrhagic enlarged liver, congested pancreas, kidneys	5/5 S-5 3/5 S-8b	S-5 OP502746 S-8b- OP502747
Broiler breeder (BB3)	10,000	Atwary, Panchagarh	8	June 2022	45/20	Drowsiness, Respiratory distress	Enlarged liver, fluid in pericardial sac, swollen kidneys with hemorrhage	5/5 S-8b	OP454908
Broiler breeder (BB4)	10,000	Atwary, Panchagarh	14	June 2022	40/15	Drowsiness, depression, respiratory distress	Hemorrhagic enlarged liver & hemorrhage at muscle & kidneys	5/5 S-8b	—
Broiler breeder (BB5)	10,000	Tetulia, Panchagarh	16	February 2021	35/12	Drowsiness, ruffled feather, sudden death	Pale enlarged liver, swollen kidneys	2/5 S-11	—
Broiler breeder (BB6)	10,000	Tetulia, Panchagarh	15	February 2021	65/30	Drowsiness, Anorexia, unable to move	Pale enlarged liver, swollen kidneys	3/5 S-11	OP454907
Commercial layer (CL1)	5,000	Dinajpur Sadar	35	November 2020	10/5	Anorexia, Reluctant to move	Enlarged liver	0/5	

^*∗*^Number of sample positive/number of sample tested. S-8b: serotype 8b, S-5: serotype 5, S-11: Serotype 11; —: sequencing not done but serotype was detected with serotype specific realtime PCR kit as mentioned in the methodology section.

**Table 2:** Detection of anti-FAdV Group 1 antibody in chicken serum by ELISA (*n* *=* 303).

Flock type	Status	Age of sampling (days)	No. of sample	Minimum/Maximum titer	Mean titer ± SE	Number/Percent seropositive	*p*value	Significance
Broiler (B1)	Beginning of exposure	9	14	211/2984	829 ± 236	2/14.28	0.019 *p* < 0.05	^*∗*^
	After exposure	35	9	1130/2502	1516 ± 128	9/100		

Broiler (B2)	Beginning of exposure	7	14	211/1285	493 ± 76	1/7.14	*p* < 0.01	^*∗∗*^
	After exposure	32	15	1488/6688	3872 ± 431	15/100		

Broiler (B3)	Beginning of exposure	11	14	139/721	475 ± 44	0/0	*p* < 0.01	^*∗∗*^
	After exposure	32	17	1367/10597	7833 ± 605	17/100		

Broiler breeder (BB1)	Beginning of exposure	14	18	115/1256	399 ± 81	2/11.11	*p* < 0.01	^*∗∗*^
	After exposure	56	15	522/2185	1246 ± 172	8/53.33		

Broiler breeder (BB2)	Beginning of exposure	9	18	215/1427	645 ± 84	3/16.66	*p* < 0.01	^*∗∗*^
	After exposure	60	16	2624/10379	6367 ± 650	16/100		

Broiler breeder (BB3)	Beginning of exposure	8	18	168/2198	667 ± 151	4/22.22	*p* < 0.01	^*∗∗*^
	After exposure	70	15	5548/14241	9699 ± 832	15/100		

Broiler breeder (BB4)	Beginning of exposure	14	15	26/1355	446 ± 111	3/20	*p* < 0.01	^*∗∗*^
	After exposure	57	15	611/2160	1452 ± 137	12/80		

Broiler breeder (BB5)	Beginning of exposure	16	15	178/1524	739 ± 121	5/33.33	*p* < 0.01	^*∗∗*^
	After exposure	57	15	791/9499	7418 ± 634	14/93.33		

Broiler breeder (BB6)	Beginning of exposure	16	15	151/2170	885 ± 172	6/40	*p* < 0.01	^*∗∗*^
	After exposure	56	15	3650/13634	9004 ± 820	15/100		

Commercial layer (CL1)	Beginning of exposure	35	15	141/393	218 ± 16	0/0	0.915 *p* > 0.05	NS
	After exposure	70	15	1/319	221 ± 14	0/0		

NS = not significant (*p* > 0.05), ^*∗*^ = significant at 5% level of probability (*p* < 0.05), ^*∗∗*^ = significant at 1% level of probability (*p* < 0.01).

## Data Availability

The data used to support the findings of this study are included within the article.

## Abbreviations

EDS: Egg drop syndrome
FAdV: Fowl adenovirus
FC: Fowl cholera
HHS: Hepatitis hydropericardium syndrome
IBD: Infectious bursal disease
IB: Infectious bronchitis
IBH: Inclusion body hepatitis
ND: Newcastle disease.
